# Integrated Proteomics and Metabolomics Analysis Reveals Potential Pathways Underlying Onion-Mediated Regulation of Spleen Immune Function in Liangshan Black Sheep

**DOI:** 10.3390/vetsci13050486

**Published:** 2026-05-17

**Authors:** Zengwen Huang, Jing Wang, Zhiqiu Huang, Gang Lv, Hehua Wang, Chaoyun Yang, Shengwang Jiang, Guiying Hao, Yi Zhang

**Affiliations:** 1 Xichang University, Xichang 615000, China; xndaxue@126.com (Z.H.);; 2 Xinjiang Taikun Group Co., Ltd., Changji 831100, China; 3 Panxi Laboratory of Animal Epidemic Disease Detection and Control of Sichuan, Xichang 615000, China; 4 Key Laboratory of Local Characteristic Goats, Xichang University, Xichang 615000, China

**Keywords:** onion, Liangshan black sheep, spleen immunity, proteomics, metabolomics

## Abstract

Feed additives play a crucial role in boosting livestock immunity, but their underlying molecular mechanisms are often incompletely understood. This study used integrated proteomics and metabolomics to systematically investigate the immunomodulatory effects of dietary fermented onion (FO) on the spleen of Liangshan Black Sheep. Results showed that FO supplementation significantly improved systemic antioxidant capacity and immune function, indicated by a higher spleen index and increased serum concentrations of SOD, MCP-1, and IL-2 (*p* < 0.05). Multi-omics profiling of spleen tissues identified 169 differentially expressed proteins and 168 differential metabolites. Integrated pathway enrichment revealed calcium signaling and purine metabolism as potential core regulatory hubs for the observed immunomodulation. This molecular remodeling correlated with key molecules including protein F2R and metabolites adenosine and hypoxanthine, which may form a coordinated regulatory network. Overall, our findings suggest potential pathways linking dietary FO supplementation to enhanced splenic immune function in Liangshan Black Sheep, likely via synergistic regulation of specific signaling pathways. This work supports FO as a promising functional feed additive and provides a molecular framework for developing novel immunomodulatory strategies in livestock production.

## Simple Summary

Feed additives improve livestock immunity, but their molecular mechanisms remain unclear. This study integrated proteomics and metabolomics to explore how fermented onion (FO) enhances splenic immunity in Liangshan Black Sheep. FO supplementation increased antioxidant and immune indices. Multi-omics revealed differential protein and metabolite profiles, highlighting calcium signaling and purine metabolism as core pathways, with F2R, adenosine, and hypoxanthine as key mediators. These findings support FO as a promising immunomodulatory feed additive.

## 1. Background

*Allium cepa* L. (onion), a cost-effective and globally cultivated crop of the Liliaceae family, is valued for its health-promoting properties attributed to organic sulfur compounds and phenolics (e.g., quercetin) [1,2,3]. These bioactives confer immunomodulatory, anti-inflammatory, and antioxidant effects, supporting its application as a livestock feed additive to improve growth performance and disease resistance [4,5,6]. Fermentation further enhances onion’s utility by hydrolyzing macromolecular conjugates to release free flavonoids (e.g., quercetin aglycone) with increased bioavailability, while reducing irritant thiosulfinates [7]. Notably, fermented plant feeds have been shown to regulate gut microbiota and enhance immune function in ruminants by activating immune cells and cytokine production [1], highlighting fermented onion (FO) as a promising functional feed ingredient. However, existing studies primarily focus on growth performance or general immune indices, lacking mechanistic insights into how FO specifically modulates immune organ function.

The spleen, as the largest secondary immune organ, plays a pivotal role in regulating innate and adaptive immunity in livestock [8,9,10]. It serves as a reservoir for lymphocytes (3/5 B cells, 2/5 T cells) and mediates pathogen clearance, cytokine secretion, and immune cell activation [8,11]. Splenic function is tightly linked to nutritional status, with dietary interventions directly influencing spleen index, immune cell populations, and signaling pathways (e.g., GH/IGF-1, IL-7R) [12,13,14]. For Liangshan Black Sheep (LBS), an indigenous breed subjected to high-altitude environmental stressors (temperature fluctuations, UV radiation, nutritional limitation), enhancing splenic immune competence via nutritional strategies is critical for sustainable livestock production [15]. Despite this, the molecular mechanisms underlying dietary FO-induced splenic immunomodulation remain elusive.

Multi-omics approaches (proteomics + metabolomics) enable systems-level dissection of functional molecules and metabolic pathways, addressing the limitations of single-omics studies [3]. While transcriptomic and metabolomic analyses have preliminarily confirmed FO’s growth-promoting effects in LBS [7], spleen-specific multi-omics investigations are lacking. This knowledge gap is significant because organ-specific gene expression patterns (as demonstrated in deer species [3]) highlight the need for targeted analysis to unravel tissue-specific regulatory networks. Moreover, quercetin—an abundant bioactive in FO—has been shown to modulate immune cell function via the Keap1/Nrf2 pathway [2,6], but its role in splenic immunomodulation in ruminants remains uncharacterized.

To fill this gap, the present study employed TMT-based quantitative proteomics and untargeted metabolomics to characterize the splenic protein and metabolite profiles of LBS fed a 20% FO-supplemented diet. The objective was to elucidate key pathways and molecular nodes underlying FO-mediated splenic immunomodulation, providing a theoretical basis for fermented plant-based feed development and precision nutrition strategies for ruminant immune health.

## 2. Materials and Methods

### 2.1. Experimental Animals

Thirty clinically healthy purebred Liangshan Black Sheep (LBS), aged approximately one month (body weight 12.00 ± 0.23 kg), were obtained from the Liangshan Black Sheep Original Breed Farm (Butuo County, Liangshan Yi Autonomous Prefecture, Sichuan Province, China). Animals were randomly assigned to two treatment groups using a random number table, each comprising three replicate pens of five sheep per pen (*n* = 15 per group). Throughout the trial, all sheep received a pelleted concentrate feed (Xinjiang Taikun Group Co., Ltd., Changji, China) at a daily allowance of 3% of body weight (dry matter basis). The control group (CON) was supplemented with corn stover at 2% of body weight, whereas the experimental group (FO) received fermented onion at an equivalent rate (2% of body weight) in place of the corn stover. All experimental animals were raised under consistent feeding and management conditions until 6 months of age. To explore the molecular mechanism underlying the differential immune phenotypes between groups, individuals with representative differential serum immune factor profiles between the control and experimental groups were selected for slaughter and tissue sample collection, avoiding artificial screening of extreme individuals.

All animal procedures were conducted at the Experimental Animal Farm of Xichang University, Xichang City, Sichuan Province, China (27°54′ N, 102°16′ E; altitude 1540 m), in strict accordance with the Guidelines for the Care and Use of Animals issued by the Animal Care Committee of Xichang University. Ethical approval was granted by the Animal Ethics Committee of Xichang University (Approval No. XCC2023003).

### 2.2. Preparation of Fermented Onion

The fermented onion (FO) used in this study was prepared in-house and was not a commercially available product. Fresh onion bulbs (*Allium cepa* L.) were manually peeled, and the apical and root portions were removed. The remaining edible tissue was sectioned into uniform cubes of approximately 1.5 cm × 1.5 cm. Sucrose (5%, *w*/*w*) and sodium chloride (1%, *w*/*w*) were added as fermentation substrates and mixed thoroughly until the onion juice was released and homogeneously distributed. The mixture was subsequently transferred into food-grade polyethylene fermentation vessels, compacted to minimize headspace, hermetically sealed, and maintained in a cool, shaded environment. Anaerobic fermentation was conducted at ambient temperature (20–25 °C) for 25 days; the containers remained unopened throughout this period to ensure sustained anaerobiosis.

Successful fermentation was confirmed by the following organoleptic criteria: the onion tissue became soft and pliable, the product emitted a characteristic sour-aromatic aroma, the color transitioned to amber-yellow, and no pungent odor or visible mold growth was detected. Chemical and microbial characterization of fermented onion was performed as follows: The final product had a pH of 3.6 ± 0.1; dominant lactic acid bacteria included *Lactobacillus plantarum*, *Lactobacillus brevis*, and *Pediococcus pentosaceus*; major bioactive compounds were quercetin (128.6 ± 7.2 mg/100 g DW), kaempferol (21.3 ± 1.5 mg/100 g DW), total organic acids (4.2 ± 0.3% DW), and short-chain fatty acids (acetic acid 1.8 ± 0.2 mg/g DW, propionic acid 0.4 ± 0.05 mg/g DW). These parameters ensured batch consistency and bioactivity stability for feeding. The detailed fermentation protocol and the physicochemical characterization of the FO product have been described in a companion study [7,10,16]. The fermented product was stored at 4 °C and consumed within 7 days of preparation for each feeding cycle.

### 2.3. Experimental Design and Dietary Treatment

Based on the preliminary observations from the 30-sheep cohort described in Section 2.1, a subset of twenty clinically healthy male LBS (aged 6 months; body weight 20.95 ± 1.43 kg) exhibiting the most pronounced inter-group divergence in serum immune factors was selected for the present proteomic and metabolomic study. Following a 14-day acclimation period during which all animals received the basal diet, the sheep were re-randomized into two groups (*n* = 5 per group) using a random number table: a control group (CON) receiving the basal diet exclusively, and a treatment group (FO20) receiving the basal diet supplemented with fermented onion at a level equivalent to 20% of the daily dietary allowance (approximately 2% of body weight). Notably, FO was provided as an additional dietary supplement on top of the complete basal ration, rather than as a replacement for any individual feed ingredient; hence, the total daily feed intake of the FO20 group comprised both the complete basal diet and the FO supplement.

The rationale for selecting the 20% FO supplementation level was derived from a preceding dose–response study conducted by the same research group [7,10,16], in which four graded inclusion levels of FO (0%, 10%, 20%, and 30% of dietary allowance) were evaluated over an identical experimental duration. The 20% FO group yielded the highest average daily gain (ADG = 248.57 g/d), significantly exceeding the control (211.43 g/d; *p* < 0.05), whereas the 30% FO group exhibited marked growth inhibition (ADG = 147.43 g/d), suggesting a dose-dependent threshold effect. The intermediate 10% group (ADG = 233.14 g/d) did not differ significantly from the control.

The basal diet was formulated according to the nutritional requirements for growing meat-type sheep specified by the National Research Council (NRC, 2007) and the Chinese Feeding Standard for Meat-Producing Sheep and Goats (NY/T 816-2021). The ingredient composition and analyzed chemical composition of the basal diet are presented in our present study; the analytical procedures for determining chemical composition followed the methodology described in a companion study [17].

All sheep were individually penned in facilities equipped with individual feeding troughs and automatic waterers. Animals were fed twice daily (08:00 and 17:00) with *ad libitum* access to clean water. The feeding trial lasted 90 days. Feed refusals were collected and weighed daily to calculate actual dry matter intake.

### 2.4. Sample Collection

At the conclusion of the 90-day feeding trial, all 20 sheep were fasted for 12 h with free access to water, weighed individually, and humanely slaughtered by exsanguination following electrical stunning, in strict accordance with the approved animal care protocol.

**Blood sampling.** Immediately prior to slaughter, jugular venous blood samples (10 mL) were collected into non-heparinized vacuum tubes. Samples were allowed to clot at ambient temperature for 30 min and subsequently centrifuged at 3000× *g* for 15 min at 4 °C. The resulting serum was aliquoted into 1.5 mL RNase-free cryovials and stored at −80 °C until analysis.

**Spleen tissue sampling.** The spleen was excised in its entirety and blotted dry with sterile absorbent paper to remove residual blood. Organ weight was recorded immediately using an analytical balance (accuracy ±0.01 g). Spleen parenchymal tissue samples were collected from the central region, mechanically homogenized on ice, aliquoted into pre-labeled 1.5 mL centrifuge tubes, snap-frozen in liquid nitrogen, and transferred to a −80 °C freezer within 30 min for long-term storage until proteomic and metabolomic analyses.

Based on the serum immune factor detection results in Section 2.6, five biologically representative individuals per group (5 individuals in the experimental group and 5 in the control group) were selected. The selected individuals well reflected the inherent inter-group differences in immune phenotypes, and were subsequently used for multi-omics profiling without deliberate pre-selection of extreme phenotypic individuals.

### 2.5. Experimental Reagents and Consumables

The principal reagents and consumables employed in this study are listed below with corresponding manufacturer information and catalogue numbers.

**Proteomic analysis reagents:** iRT Kit (Biognosys AG, Schlieren, Switzerland); Bradford Protein Quantification Kit (Beyotime Biotechnology, Shanghai, China); sodium dodecyl sulfate (SDS; Sinopharm Chemical Reagent Co., Ltd., Shanghai, China); mass spectrometry-grade trypsin (Promega Corporation, Madison, WI, USA; Cat. No. V5280); LC-MS-grade acetonitrile (Thermo Fisher Scientific, Waltham, MA, USA; Cat. No. A955-4); High-Select^TM^ Top14 Abundant Protein Depletion Mini Spin Columns (Thermo Fisher Scientific; Cat. No. A36370); formic acid (Fluka, Buchs, Switzerland); protease inhibitor cocktail III, TMT 10-plex Isobaric Mass Tagging Kit, acetonitrile, and ultrapure water (all from Thermo Fisher Scientific, Waltham, MA, USA); 2-D Quant Kit (Cytiva, formerly GE Healthcare, Buckinghamshire, UK).

**Additional chemical reagents (Sigma-Aldrich, St. Louis, MO, USA):** ammonium bicarbonate, iodoacetamide (IAA), dithiothreitol (DTT), trifluoroacetic acid (TFA), ethylenediaminetetraacetic acid (EDTA), urea, and tetraethylammonium bromide (TEAB).

**ELISA kits:** Sheep-specific ELISA kits for immunoglobulin A (IgA), immunoglobulin G (IgG), immunoglobulin M (IgM), complement component 3 (C3), superoxide dismutase (SOD), monocyte chemoattractant protein-1 (MCP-1), and interleukin-2 (IL-2) were procured from Shanghai Enzyme-linked Biotechnology Co., Ltd. (Shanghai, China).

### 2.6. Determination of Serum Immune Factors

Serum concentrations of IgA, IgG, IgM, C3, SOD, MCP-1, and IL-2 were quantified using the respective sheep-specific sandwich ELISA kits (Shanghai Enzyme-linked Biotechnology Co., Ltd., Shanghai, China). All assays were performed strictly in accordance with the manufacturer’s instructions. Briefly, serum samples and standard solutions were added to pre-coated 96-well microplates and incubated at 37 °C for 30 min. After five consecutive wash cycles, enzyme-conjugated detection antibody was added and incubated under identical conditions. Following a second wash cycle, TMB chromogenic substrate was added and the reaction was terminated with the supplied stop solution. Optical density was measured at 450 nm using a microplate reader within 15 min of termination. Each sample was assayed in triplicate, and concentrations were calculated from the respective standard curves. Inter-assay and intra-assay coefficients of variation were maintained below 10% and 8%, respectively.

### 2.7. Determination of Spleen Index in Liangshan Black Sheep

After recording live body weight and spleen weight, the spleen index was calculated using the following formula:

Spleen index = spleen weight (g)/body weight (kg) × 100%.

The spleen index serves as a standardized morphometric indicator reflecting the relative development and functional status of the spleen in relation to overall body size [18].

### 2.8. Protein Extraction, Quantification, and Enzymatic Hydrolysis

Based on the serum immune factor determinations (Section 2.6), spleen tissue specimens from animals exhibiting highly significant inter-group differences in immune indicators (*p* < 0.01) were selected for proteomic analysis (*n* = 5 per group).

**Protein extraction.** Frozen spleen tissue (~100 mg) was retrieved from −80 °C storage, cryogenically pulverized under liquid nitrogen, and transferred to pre-cooled centrifuge tubes. An appropriate volume of SDT lysis buffer (4% SDS, 100 mM Tris-HCl, pH 7.6) supplemented with 100 mM NaCl and DTT solution (1:100 *v*/*v*) was added. The homogenate was vortexed briefly, sonicated in an ice-water bath for 5 min (pulse: 5 s on/10 s off), heated at 95 °C for 8–15 min for protein denaturation, cooled on ice for 2 min, and centrifuged at 12,000× *g* for 15 min at 4 °C. The supernatant was collected and incubated with IAA solution in the dark at room temperature for 1 h to achieve alkylation of cysteine residues. Four volumes of pre-cooled acetone (−20 °C) were subsequently added, and proteins were precipitated at −20 °C for ≥2 h. The precipitate was recovered by centrifugation (12,000× *g*, 15 min, 4 °C), washed once with 1 mL of pre-cooled acetone (−20 °C), air-dried, and reconstituted in DB dissolution buffer (8 M urea, 100 mM TEAB, pH 8.5).

**Protein quantification.** Protein concentration was determined using the Bradford Protein Quantification Kit (Beyotime Biotechnology, Shanghai, China) following the manufacturer’s protocol. A standard curve was generated using bovine serum albumin (BSA) over a concentration range of 0–0.5 μg/μL. Aliquots (20 μL) of standard and diluted sample solutions were dispensed into a 96-well plate in triplicate, followed by the addition of 180 μL of Coomassie Brilliant Blue G-250 reagent. After incubation at room temperature for 5 min, absorbance was measured at 595 nm. Protein concentrations of unknown samples were interpolated from the standard curve.

**SDS-PAGE verification.** Twenty micrograms of protein from each sample were resolved on 12% SDS-PAGE gels (stacking gel: 80 V, 20 min; resolving gel: 120 V, 90 min). Gels were stained with Coomassie Brilliant Blue R-250 and destained until discrete protein bands were clearly visible, confirming protein integrity and absence of degradation.

**Enzymatic hydrolysis.** Protein samples were diluted in DB lysis buffer to a final volume of 100 μL, and trypsin (mass spectrometry-grade; Promega, Cat. No. V5280) was added at a 1:50 (*w*/*w*) enzyme-to-substrate ratio in the presence of 100 mM TEAB buffer. Digestion was performed at 37 °C for 4 h. Additional trypsin and 1 mM CaCl_2_ were subsequently added, and the digestion was continued overnight (~16 h) at 37 °C. The reaction was quenched by acidification with formic acid to pH < 3. The digest was centrifuged at 12,000× *g* for 5 min at room temperature, and the supernatant was desalted using a C18 solid-phase extraction cartridge (washed three times with 0.1% formic acid in water). Peptides were eluted with 70% acetonitrile containing 0.1% formic acid, lyophilized by vacuum centrifugation, and stored at −80 °C until LC-MS/MS analysis.

### 2.9. LC-MS/MS Analysis and Proteomic Data Processing

**Liquid chromatography**. Lyophilized peptides were reconstituted in 0.1% (*v*/*v*) formic acid (mobile phase A) and separated using a NanoElute ultra-high-performance liquid chromatography (UHPLC) system (Bruker Daltonics, Bremen, Germany). Mobile phase B consisted of acetonitrile containing 0.1% (*v*/*v*) formic acid. Separation was achieved using the following gradient: 6–22% B over 0–70 min, 22–32% B over 70–84 min, 32–80% B over 84–87 min, and 80% B maintained from 87 to 90 min. The flow rate was set at 300 nL/min.

**Mass spectrometry.** Eluted peptides were introduced into a CaptiveSpray nanoelectrospray ionization source operated at 1.4 kV and analyzed using a timsTOF Pro mass spectrometer (Bruker Daltonics, Bremen, Germany) equipped with a time-of-flight (TOF) detector. The scan range for both precursor and fragment ions was set at 100–1700 *m/z*. Data were acquired in parallel accumulation–serial fragmentation (PASEF) mode. Each MS1 scan was followed by 10 PASEF MS/MS scans targeting precursor ions with charge states 0–5. Dynamic exclusion was applied with a window of 24 s to minimize redundant fragmentation of previously sampled precursors.

**Database searching and protein identification.** Raw data files were processed using DIA-NN software (version 1.8) with the *Ovis aries* reference proteome database (ARS-UI_Ramb_v3.0; 76,699 sequences). The search parameters were configured as follows: precursor ion mass tolerance, 10 ppm; fragment ion mass tolerance, 0.02 Da; fixed modification, cysteine carbamidomethylation; variable modifications, methionine oxidation, N-terminal acetylation, methionine loss, and methionine loss + acetylation; maximum missed cleavage sites, 1. The results were filtered to retain only peptide-spectrum matches (PSMs) and corresponding proteins meeting a ≥99% confidence threshold. A false discovery rate (FDR) of ≤1% was applied at both the peptide and protein levels.

**Functional annotation.** Identified proteins were subjected to Gene Ontology (GO) and InterPro (IPR) functional annotation using InterProScan software (version 5.62-94.0; EMBL-EBI, Hinxton, UK) in conjunction with the Pfam and PRINTS databases. Protein families and signaling pathways were classified using the Clusters of Orthologous Groups (COG) database and the Kyoto Encyclopedia of Genes and Genomes (KEGG; https://www.kegg.jp/). Differentially expressed proteins (DEPs) were subjected to volcano plot visualization, hierarchical clustering heatmap analysis, and GO/IPR/KEGG pathway enrichment analysis. Potential protein–protein interactions (PPIs) were predicted using the STRING database (version 11.0; http://string.embl.de/) with a minimum interaction confidence score of 0.4.

### 2.10. Metabolite Extraction from Spleen Tissue

Spleen tissue specimens selected for metabolomic analysis (Section 2.4) were retrieved from −80 °C storage. Approximately 50 mg of tissue was accurately weighed into a pre-cooled 2 mL centrifuge tube, and 1000 μL of extraction solvent (methanol:acetonitrile: water = 2:2:1, *v*/*v*/*v*, pre-cooled to −20 °C) was added. The mixture was vortexed for 30 s, ground using a cryogenic tissue grinder (frequency: 35 Hz, 5 min), and subjected to ultrasonication in an ice-water bath for 5 min. The resulting homogenate was incubated at −40 °C for 1 h to facilitate protein precipitation. Subsequently, the extract was centrifuged at 10,000 rpm for 15 min at 4 °C. The supernatant (825 μL) was carefully transferred to a clean centrifuge tube and evaporated to dryness under vacuum.

The dried residue was reconstituted in 200 μL of 50% acetonitrile (*v*/*v*, aqueous), vortexed for 30 s, sonicated in an ice-water bath for 10 min, and centrifuged at 13,000 rpm for 15 min at 4 °C. The supernatant (75 μL) was transferred to a chromatographic sample vial fitted with a glass insert for LC-MS analysis. The remaining supernatant was stored at −80 °C as a backup.

**Quality control (QC) sample preparation**. A pooled QC sample was prepared by combining equal aliquots (10 μL) of the supernatant from each individual sample. The QC sample (75 μL per injection) was analyzed at regular intervals throughout the analytical sequence to monitor instrument stability and data quality.

### 2.11. LC-MS/MS-Based Metabolite Identification and Data Processing

**Data acquisition.** Raw spectral data (.raw files) were imported into Compound Discoverer 3.3 (CD 3.3; Thermo Fisher Scientific, Waltham, MA, USA). Peaks were extracted, quantified, and subjected to target ion integration using the following parameters: mass deviation ≤ 5 ppm and signal intensity deviation ≤ 30%. The initial QC sample was used for peak area calibration to improve identification accuracy. Molecular formulae were predicted based on molecular ion peaks and fragment ion patterns and compared against the reference database. Background ions were removed by blank subtraction, and the resulting quantitative data were normalized to relative peak areas. Metabolite features with a coefficient of variation (CV) > 30% in QC samples were excluded from further analysis.

All data processing was performed on a Linux platform (CentOS 6.6; Red Hat, Inc., Raleigh, NC, USA) using R (version 4.2.1; R Core Team, Vienna, Austria) and Python (version 3.8.10; Python Software Foundation).

**Metabolite annotation and multivariate statistical analysis.** Identified metabolites were annotated against the KEGG (https://www.kegg.jp/), HMDB (version 5.0; https://hmdb.ca/), and LIPID Maps (https://www.lipidmaps.org/) databases. Following data transformation using metaX software (version 1.0; Peking University, Beijing, China), the dataset was subjected to principal component analysis (PCA) and partial least-squares discriminant analysis (PLS-DA) to obtain variable importance in projection (VIP) scores. Univariate significance testing was performed using the independent-samples Student’s *t*-test.

**Differential metabolite screening.** Metabolites were classified as differentially expressed when meeting the dual criteria of VIP > 1 and *p* < 0.05. KEGG pathway enrichment analysis was subsequently performed on the differential metabolites. Pathways were considered significantly enriched when *p* < 0.05 and the ratio of enriched metabolites to total identified metabolites exceeded 1/10.

### 2.12. Integrated Analysis of Proteomics and Metabolomics Data

The differentially expressed proteins and metabolites identified in Section 2.9 and Section 2.11, respectively, were subjected to integrated pathway mapping to identify co-regulated biological processes. Both datasets were mapped to the KEGG pathway database using the *Ovis aries* reference proteome (ARS-UI_Ramb_v3.0; 76,699 sequences). Concordance between proteomic and metabolomic expression patterns within shared pathways was assessed, with particular emphasis on metabolic intermediates and their cognate enzymes.

Upstream and downstream metabolite–enzyme relationships within immune-relevant anabolic pathways were traced according to established metabolic principles to identify candidate regulatory nodes. Correlation analysis between key proteins and metabolites was performed using MetaboAnalyst (version 5.0; https://www.metaboanalyst.ca/). Protein–protein interaction networks were constructed and visualized using Cytoscape (version 3.6.1; National Resource for Network Biology, San Diego, CA, USA), and overlapping differential features were identified using Venny (version 2.1; BioinfoGP, CNB-CSIC, Madrid, Spain).

An integrated molecular network depicting the regulatory relationship between fermented onion supplementation and splenic immune-related protein factors and metabolic immune molecules was constructed using Cytoscape and R (version 4.2.1) based on the multi-omics screening results, following established biochemical topology and metabolic pathway architecture.

### 2.13. Statistical Analysis

Prior to all inferential analyses, the normality of data distribution was assessed using the Shapiro–Wilk test (*shapiro.test* function in R, version 4.2.1), and homogeneity of variances was evaluated using Levene’s test (*leveneTest* function, *car* package in R). Datasets satisfying both assumptions (*p* > 0.05 for the Shapiro–Wilk and Levene’s tests) were subjected to parametric testing. Inter-group comparisons between CON and FO were performed using the independent-samples Student’s *t*-test (*t.test* function in R).

Statistical power was evaluated *a priori* using the *pwr* package in R (version 1.3-0), with an assumed large effect size (Cohen’s *d* = 0.8), a significance level of α = 0.05, and a target power of 1 − β = 0.80.

All results are expressed as mean ± standard error of the mean (SEM). A threshold of *p* < 0.05 was considered statistically significant, and *p* < 0.01 was considered highly significant. All statistical analyses were performed using R (version 4.2.1; R Core Team, Vienna, Austria).

## 3. Results

### 3.1. Effect of Fermented Onion on the Contents of Immune Factors and Antioxidant-Related Factors in the Blood of Liangshan Black Sheep

The present study employed one-way analysis of variance to investigate the effect of fermented onion on the contents of immune factors and antioxidant-related factors in the blood of Liangshan Black Sheep. The study compared and analyzed the differences in immune factors and antioxidant-related factor indices in the spleen tissue between the experimental group (T) and the control group (CON). As demonstrated in Table 1, the levels of immunoglobulin A (IgA), immunoglobulin G (IgG), immunoglobulin M (IgM), and complement 3 (C3) in the serum of Liangshan Black Sheep in the experimental group were all numerically elevated relative to those in the control group, but these differences did not reach statistical significance (*p* > 0.05). This observation suggests a trend toward enhanced humoral immune component synthesis, which may require a longer feeding duration or larger sample size to achieve statistical significance. In contrast, the levels of superoxide dismutase (SOD), monocyte chemoattract protein-1 (MCP-1), and interleukin-2 (IL-2) were significantly higher in the experimental group than in the control group (*p* < 0.05). FO supplementation rapidly improves systemic antioxidant capacity and promotes immune cell activation, chemotaxis, and proliferation—key early events in immune enhancement—whereas increases in immunoglobulins may represent a later, cumulative adaptive response.

### 3.2. Effect of Fermented Onion on the Spleen Index of Liangshan Black Sheep

The present study set out to investigate the effect of fermented onion on the spleen index of the Liangshan Black Sheep. To this end, Liangshan Black Sheep with significant differences in serum immune-related factors were selected for slaughter experiments. The spleen was swiftly extracted and weighed in a sterile environment, with the resulting data presented in Table 2. A thorough examination of the data presented in Table 2 has revealed a statistically significant increase in the spleen index of the experimental group in comparison with the control group (CON group). This increase was found to be highly significant at the 0.05 level of probability. This finding suggests that the experimental group can effectively increase the spleen index.

### 3.3. Identification of Spleen Protein Sequences in Liangshan Black Sheep by DIA Technology

**Figure 1:**
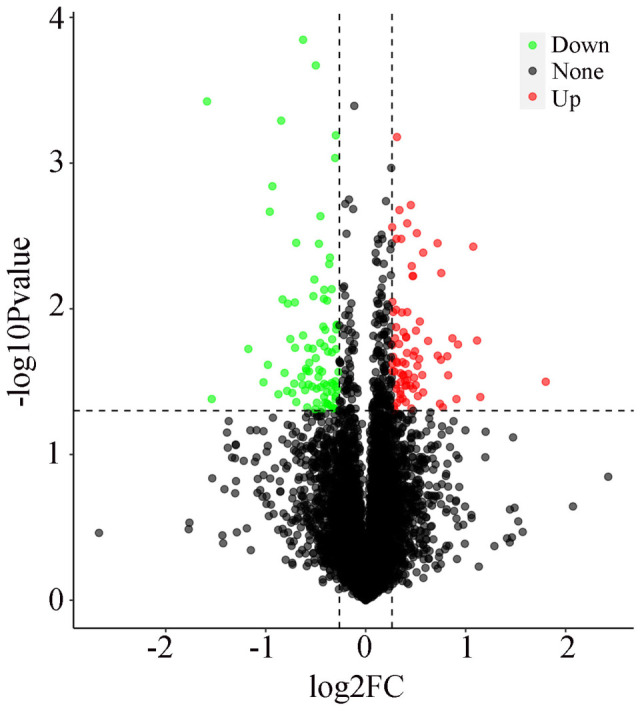
Volcano plot of differentially expressed proteins in spleen tissue between the FO and CON groups.

In this study, DIA quantitative proteomics technology was utilized for the sequencing analysis of the spleen tissue of the Liangshan Black Sheep. The “242,2658.fasta (76,699 sequences)” database was utilized to identify the protein sequences in 10 spleen samples of Liangshan Black Sheep (five in the control group and five in the experimental group). The study identified a total of 139,741 peptides from the 10 spleen tissue samples of Liangshan Black Sheep, with the length of these peptides primarily distributed between 7 and 30 amino acid residues (Supplementary Figure S1A). In addition, protein sequences in each sample were identified in accordance with the standard of “DIA-NN analysis parameters: It is imperative to note that both Global.Q.Value (global precursor q-value) and Global.Q.Value (global q-value for the protein group) must be less than 0.01. The results demonstrated that the number of protein sequences identified in each sample exceeded 9600. However, there were certain discrepancies in the types and quantities of proteins between the groups. The specific results are presented inFigure S1B. A subsequent comprehensive analysis of the protein sequences present in all samples revealed the identification of a total of 9974 protein sequences from the 10 Liangshan Black Sheep spleen tissue samples (Figure 1).

### 3.4. Screening and Identification of Differentially Expressed Proteins in Spleen Tissue Between Experimental Group and Control Group of Liangshan Black Sheep

The analysis revealed 169 proteins that exhibited statistically significant differences between the spleen tissue of the Liangshan Black Sheep in the experimental group and the control group. The screening criteria for upregulated proteins were FC > 1.2 and *p* < 0.05, while for downregulated proteins, the criteria were FC < 0.83 and *p* < 0.05. A comparison of the experimental and control groups revealed that 82 proteins in the spleen tissue of the Liangshan Black Sheep showed an increased expression, while 87 proteins demonstrated a decreased expression. The visual analysis of the expression levels of differentially expressed proteins is illustrated in Figure 1.

### 3.5. Bioinformatics Analysis of Differentially Expressed Proteins in Spleen Tissue Between Experimental Group and Control Group of Liangshan Black Sheep

**Figure 2:**
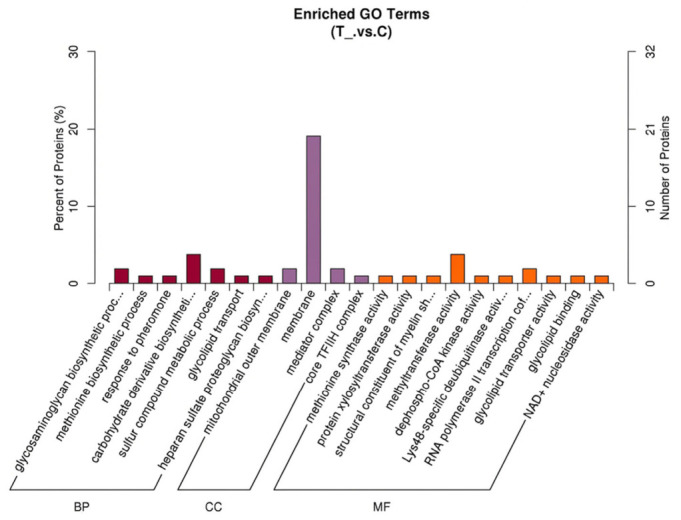
GO enrichment analysis of differentially expressed proteins in spleen tissues of Liangshan black sheep between the experimental group and the control group.

GO annotation analysis was performed on the 169 proteins exhibiting diverse expression patterns. The results demonstrated that among the differentially expressed proteins, a mere 105 could be annotated to 144 GO terms, including 67 terms enriched in biological process (BP), 21 in cellular component (CC), and 56 in molecular function (MF). Subsequent screening of proteins enriched in GO terms according to the standard of *p* < 0.05 revealed that there were 7 enriched terms in BP, 4 in CC, and 10 in MF that met the criteria. The number of proteins enriched in the “membrane” term was the largest of all (20 proteins), as illustrated in Figure 2.

**Figure 3:**
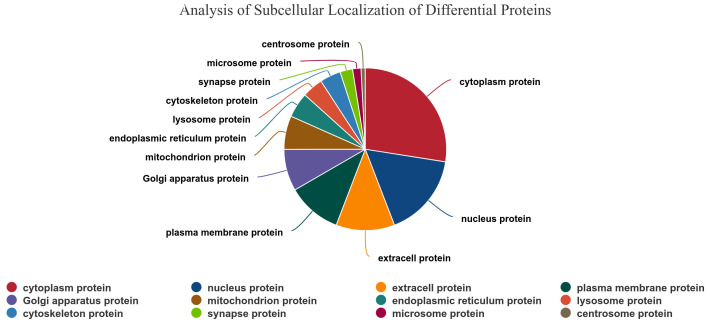
Differentially expressed proteins in spleen tissues of Liangshan black sheep between the experimental group and the control group in subcellular localization analysis.

Concurrently, subcellular localization analysis was conducted on the 169 differentially expressed proteins. The results demonstrated that the differential proteins were predominantly localized in subcellular structures, including the cytoplasm, mitochondria, nucleus, extracellular space, cytoplasmic nucleus, and plasma membrane. The predominant category was that of cytoplasmic proteins, which accounted for 27.50% of the total, as illustrated in Figure 3.

**Figure 4:**
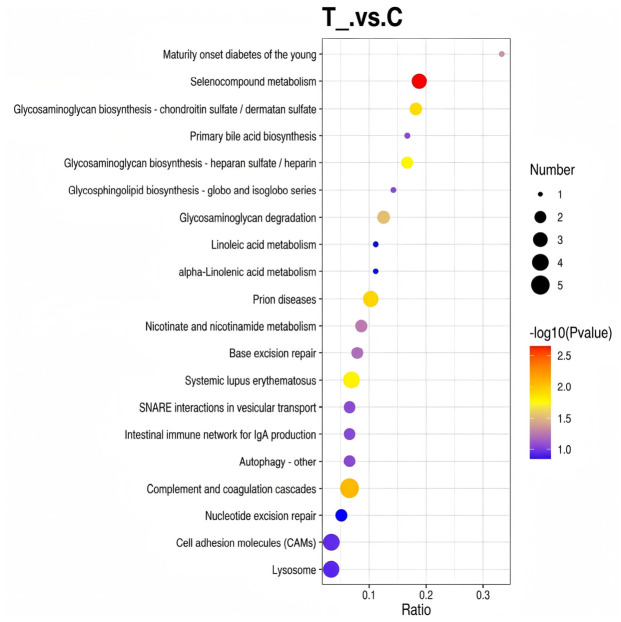
KEGG annotation analysis of differentially expressed proteins in spleen tissues of Liangshan black sheep between the experimental group and the control group.

An annotation analysis of the 169 proteins was conducted using the KEGG database. The results demonstrated that among the differential proteins, only 74 could be annotated to 99 KEGG biological pathways, of which 19 were enriched in the “Metabolic pathways” pathway. Subsequent KEGG enrichment analysis was conducted for each comparison pair, with the results of pathways significantly enriched by differentially expressed proteins illustrated in Figure 4.

### 3.6. Identification of Spleen Metabolites in Liangshan Black Sheep by Liquid Chromatography–Mass Spectrometry (LC-MS) Technology

**Figure 5:**
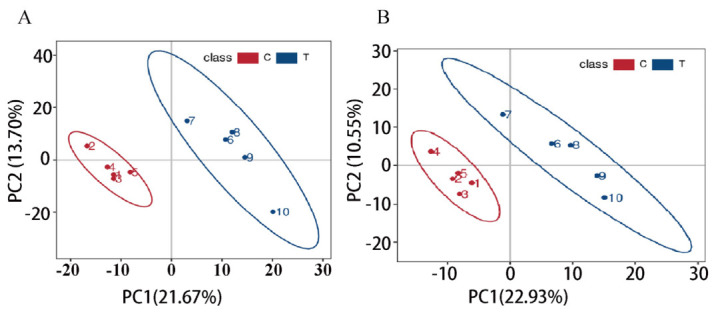
PLS-DA analysis of spleen metabolites in Liangshan Black Sheep (FO vs. CON). Note: (**A**) Positive ion mode; (**B**) Negative ion mode.

In this study, untargeted metabolomics technology was utilized for the sequencing analysis of the spleen tissue of the Liangshan Black Sheep, and the metabolite sequences in 10 spleen samples of the Liangshan Black Sheep were identified. The results demonstrated that a total of 1411 metabolites could be identified from the 10 spleen tissue samples of Liangshan Black Sheep, including 896 metabolites in positive ion mode and 515 metabolites in negative ion mode. The 1411 identified metabolites were then subjected to partial least squares discriminant analysis (PLS-DA). The results demonstrated that there were significant inter-group differences in the metabolites present in the spleen tissue of the Liangshan Black Sheep in both the positive and negative ion modes, as illustrated in Figure 5A,B.

### 3.7. Screening and Identification of Differential Metabolites in Spleen Tissue Between Experimental Group and Control Group of Liangshan Black Sheep

**Figure 6:**
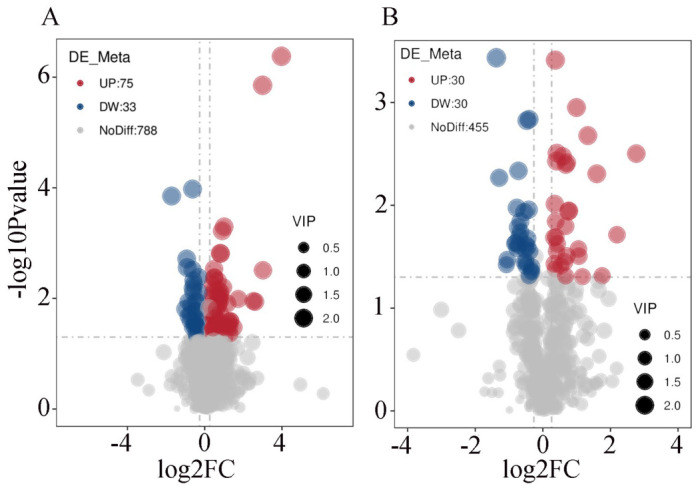
Volcano plot visualization analysis of differential metabolites in spleen tissues of Liangshan black sheep between the experimental group and the control group. Note: (**A**) represents the visual analysis of differential metabolites between the test group and the control group in positive ion mode; (**B**) represents the visual analysis of differential metabolites between the test group and the control group in negative ion mode.

In order to further analyze the specific differences in metabolites between the two groups in positive and negative ion modes, this study screened the 1411 differential metabolites identified in the two groups according to the set threshold (VIP > 1.0, FC > 1.2 or FC < 0.833, and *p*-value < 0.05). The results demonstrated that in positive ion mode, there were 108 significantly differential metabolites between the two groups, among which 75 were found to be overexpressed and 33 underexpressed in the experimental group; in negative ion mode, there were 60 significantly differential metabolites between the two groups, among which 30 were overexpressed and 30 underexpressed in the experimental group. The results of the visual analysis of differential metabolites are illustrated in Figure 6A,B.

### 3.8. Bioinformatics Analysis of Differential Metabolites in Spleen Tissue Between Experimental Group and Control Group of Liangshan Black Sheep

**Figure 7:**
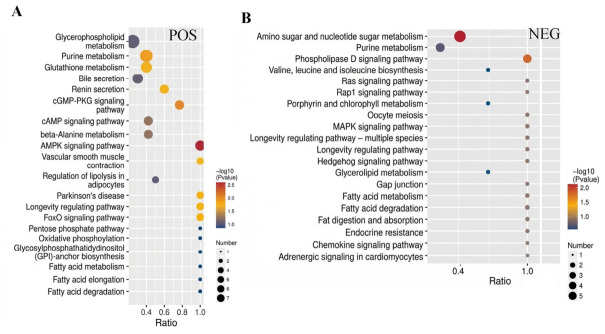
KEGG analysis of differential metabolites in spleen tissue of Liangshan black sheep between the experimental group and the control group. Note: (**A**) represents the differential metabolites between the experimental group and the control group in positive ion mode obtained from KEGG analysis, (**B**) represents the differential metabolites between the experimental group and the control group in negative ion mode obtained from KEGG analysis.

The Kyoto Encyclopedia of Genes and Genomes (KEGG) was utilized for further analysis of the differential metabolites in the spleen tissue of Liangshan Black Sheep between the experimental group and the control group. The results demonstrated that among the 108 differential metabolites in positive ion mode, a mere 29 could be annotated to 76 pathways in KEGG. Of these, the largest number of metabolites annotated to the “Metabolic pathways” pathway was 23. Similarly, among the 60 differential metabolites in negative ion mode, only 21 could be annotated to 44 pathways in KEGG, with the “Metabolic pathways” pathway accounting for the largest number of metabolites (15). The visual analysis of metabolites annotated to KEGG metabolic pathways is illustrated in Figure 7A,B.

### 3.9. Integrated Analysis of Differentially Expressed Proteins and Metabolites in Spleen Tissue Between Experimental Group and Control Group of Liangshan Black Sheep

As indicated by the results of the proteomics analysis, of the 169 differentially expressed proteins identified in the spleen tissue of the Liangshan Black Sheep population between the experimental and control groups, 74 could be annotated to 99 KEGG pathways. As indicated by the metabolomics analysis results, in positive ion mode, 29 out of 108 differentially expressed metabolites could be annotated to 76 KEGG pathways; in negative ion mode, 21 out of 60 differential metabolites could be annotated to 44 KEGG pathways.

**Figure 8:**
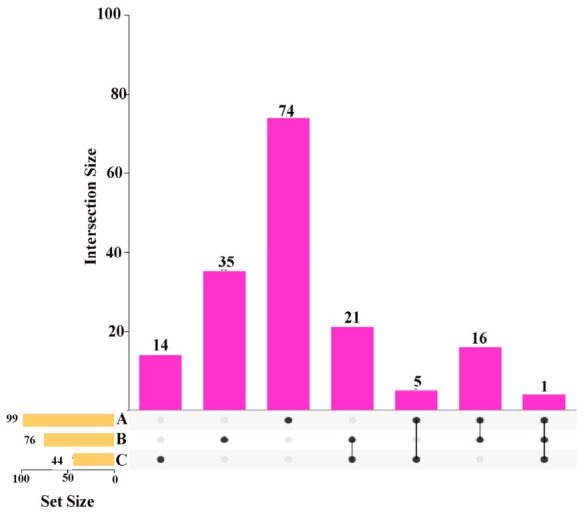
Integrated analysis of differentially expressed proteins and metabolites in spleen tissues of Liangshan black sheep between the experimental group and the control group.

Intersection analysis was performed on the KEGG pathways annotated by differentially expressed proteins and metabolites in the spleen tissue of the two groups. The results demonstrated that there were 20 KEGG pathways co-enriched by differentially expressed proteins and differential metabolites in positive ion mode; 9 KEGG pathways co-enriched by differentially expressed proteins and differential metabolites in negative ion mode; and 4 KEGG pathways co-enriched by differentially expressed proteins and differential metabolites in both positive and negative ion modes (“Calcium signaling pathway”, “Purine metabolism”, “Metabolic pathways”, and “Phospholipase D signaling pathway”), as illustrated in Figure 8. A total of 28 proteins and 52 metabolites were identified, of which 31 were annotated in positive ion mode and 21 in negative ion mode. This integrated analysis shows correlative associations, not definitive causal links between calcium signaling, purine metabolism, and splenic immune function. These pathways are highlighted as core regulatory hubs based on co-enrichment and differential expression, but direct causal links remain to be validated by functional experiments.

### 3.10. Interaction Analysis of Differentially Expressed Proteins and Metabolites in Spleen Tissue Between Experimental Group and Control Group of Liangshan Black Sheep

**Figure 9:**
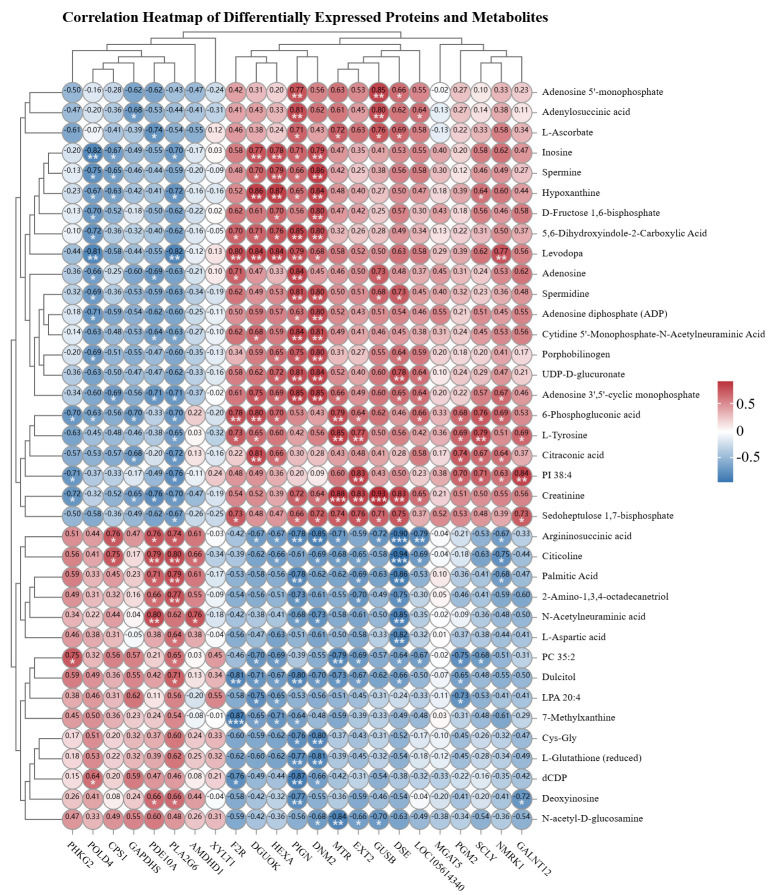
Correlation analysis of differentially expressed proteins and metabolites in spleen tissues of Liangshan black sheep between the experimental group and the control group. The asterisk Indicates statistical significance, * *p* < 0.05, ** *p* < 0.01，*** *p* < 0.001.

Subsequent to the integration analysis of differentially expressed proteins and metabolites in the spleen tissue of Liangshan Black Sheep between the experimental group and the control group, further correlation and interaction analyses of proteomics and metabolomics data were conducted. A subsequent investigation into the dynamic correlation heatmap analysis of the two sets of data demonstrated that the majority of differentially expressed proteins exhibited significant correlations with metabolites. The visual characterization of differentially expressed proteins and metabolites based on correlation strength is demonstrated in Figure 9.

**Figure 10:**
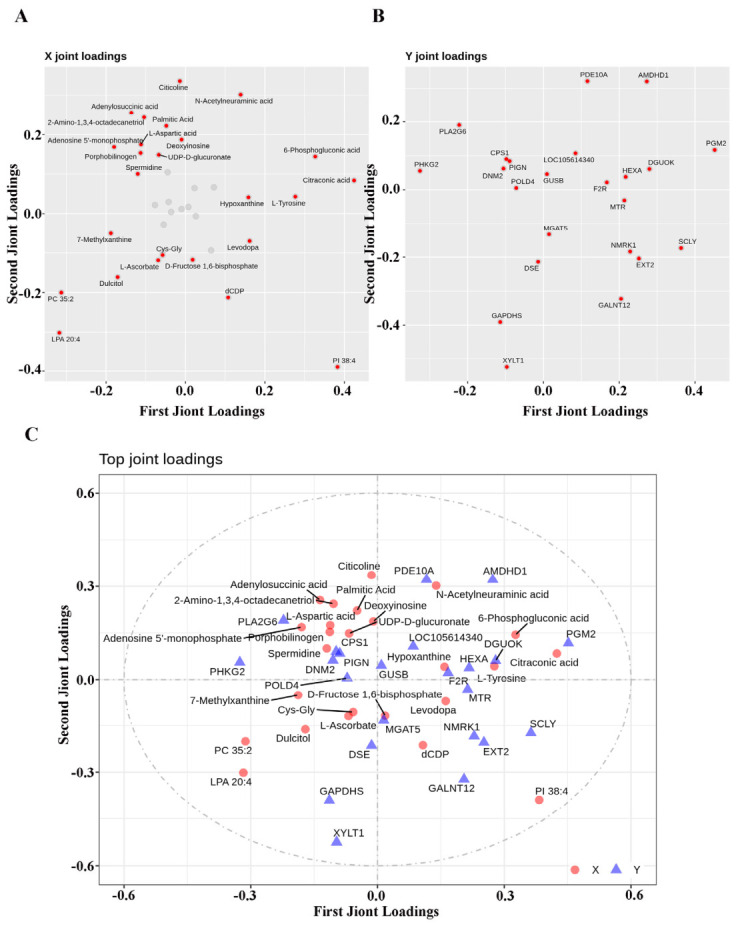
O2PLS correlation analysis of differentially expressed proteins and metabolites in spleen tissues of Liangshan black sheep between the experimental group and the control group. Note: (**A**) represents the loading plot of differentially expressed proteins in spleen tissue of Liangshan black sheep between the experimental group and the control group analyzed by O2PLS; (**B**) represents the loading plot of differentially expressed metabolites in spleen tissue of Liangshan black sheep between the experimental group and the control group analyzed by O2PLS; (**C**) represents the loading plot of the correlation between differentially expressed proteins and metabolites in spleen tissue of Liangshan black sheep between the experimental group and the control group analyzed by O2PLS.

In addition, the orthogonal partial least squares (O2PLS) method was employed for bidirectional orthogonal partial least squares modeling and prediction of differentially expressed proteins and metabolites to reveal their intrinsic associations, thereby quantifying the integration degree of the two omics data and identifying key proteins and metabolites that dominate this association. The results of the analysis are presented in Figure 10A, Figure 10B and Figure 10C, respectively. The present interaction analysis successfully identified protein–metabolite combinations that exhibited a high degree of correlation. This finding will inform future research directions, with a particular focus on the in-depth analysis of the multi-omics association mechanism in subsequent studies.

### 3.11. Screening and Identification of Differentially Expressed Immune-Related Proteins and Metabolites in Spleen Tissue Between Experimental Group and Control Group of Liangshan Black Sheep

A detailed investigation, underpinned by KEGG functional annotation, revealed that among the 28 proteins and 52 metabolites that exhibited differential expression in the spleen tissue of Liangshan Black Sheep between the experimental group and the control group, five proteins (F2R, PLA2G6, PIGN, SCLY, MGAT5) and 10 metabolites (adenosine 3′,5′-cyclic monophosphate, adenosine, hypoxanthine, L-glutathione (reduced), L-ascorbate, spermidine, spermine, L-tyrosine, L-aspartic acid, citicoline) were found to be directly associated with the immune function of the body.

Further analysis revealed that among the five proteins, four were found to be upregulated in the spleen tissue of the Liangshan Black Sheep in the experimental group compared with the control group (F2R, PIGN, SCLY, MGAT5), while one protein (PLA2G6) was downregulated. Among the ten metabolites, seven were found to be overexpressed in the spleen tissue of the Liangshan Black Sheep in the experimental group in comparison with the control group (adenosine 3′,5′-cyclic monophosphate, adenosine, hypoxanthine, L-ascorbate, spermidine, spermine, L-tyrosine), while three metabolites (L-glutathione (reduced), L-aspartic acid, citicoline) were found to be underexpressed. The specific functional information of the five immune-related proteins and ten immune-related metabolites is shown in Table 3.

### 3.12. Molecular Network Interaction Analysis of Differentially Expressed Immune-Related Proteins and Metabolites in Spleen Tissue Between Experimental Group and Control Group of Liangshan Black Sheep

Life activities are complex biological processes. It is evident that a solitary protein or enzyme is incapable of exhibiting biological activity in isolation, with specific functions only being realized through the concerted action of mutual regulation. Furthermore, the analysis of their signal transduction mechanisms cannot be conducted exclusively through two-dimensional maps. The present study utilized the STRING and Cytoscape software programmes to construct a molecular interaction network, with the objective of elucidating the interaction relationships between the 15 differentially expressed immune-related proteins and metabolites that were screened from the spleen tissue of Liangshan Black Sheep. These proteins and metabolites were compared between the experimental group and the control group.

**Figure 11:**
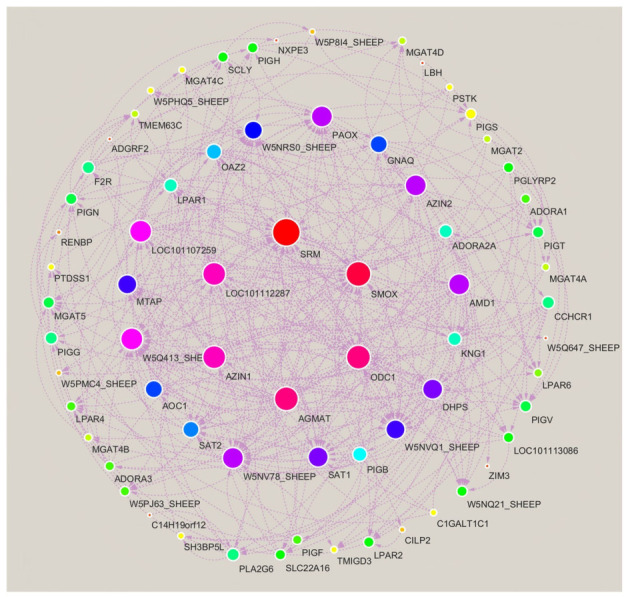
Molecular network interaction analysis of differentially expressed immune proteins and metabolites in spleen tissues of Liangshan black sheep between the experimental group and the control group.

The molecular interaction network (Figure 11) illustrates potential connections among immune-related proteins and metabolites; however, this network is visually complex and derived from in silico prediction only. It lacks quantitative validation and may overrepresent indirect or low-confidence associations. Most interactions are indirect; metabolites with no clear network links are not non-functional—they may act via uncharacterized pathways or weaker interactions not captured by this analysis.

## 4. Discussion

Liangshan Black Sheep represents a genetically valuable indigenous ruminant breed in southwest China, where immune competence directly underpins growth performance, health resilience, and sustainable production. Onion (*Allium cepa* L.) is rich in flavonoids, organosulfur compounds, and other bioactive constituents known to modulate animal immunity, with fermentation further enhancing the bioavailability of quercetin and other functional components [17,18,19,20]. As the largest peripheral immune organ, the spleen governs central processes of innate and adaptive immunity, and spleen index serves as a reliable biomarker for immune organ development and systemic immune status [19,21]. Previous studies reported that quercetin promotes splenocyte proliferation and elevates spleen index in livestock [22]. In the present study, dietary supplementation with fermented onion (FO) significantly increased spleen index (*p* < 0.05), providing a foundational phenotypic basis for investigating molecular mechanisms underlying FO-mediated immunomodulation.

Onion-derived polyphenols contribute strongly to antioxidant capacity, which protects lymphocytes and macrophages from oxidative stress and preserves immune cell function [23,24,25,26]. Quercetin has been shown to activate the Nrf2/HO-1 antioxidant pathway, enhance IL-2 and IL-6 secretion, and boost macrophage phagocytic activity in avian and murine models [27,28]. Consistent with these reports, the present study demonstrated that FO supplementation significantly elevated serum levels of SOD, MCP-1, and IL-2 (*p* < 0.05), whereas IgA, IgG, and IgM exhibited non-significant upward trends. These observations support an early-phase immune activation pattern induced by FO: antioxidant defense and immune cell recruitment are rapidly enhanced, whereas immunoglobulin synthesis and humoral maturation may require a longer feeding duration to achieve statistical significance. This temporal distinction provides a biologically plausible explanation for the observed immunoglobulin profiles and reinforces the conclusion that FO enhances splenic immune function through coordinated antioxidant and immunostimulatory actions.

To delineate the molecular basis of splenic immunomodulation by FO, we performed an integrated proteomic and metabolomic profiling. In total, 169 differentially expressed proteins and 168 differential metabolites were identified, from which 9 immune-associated molecules were prioritized: 5 proteins (F2R, PLA2G6, PIGN, SCLY, MGAT5) and 4 metabolites (adenosine, hypoxanthine, spermidine, spermine). Pathway enrichment revealed convergence on Metabolic pathways (ko01100), Calcium signaling, and Purine metabolism, supporting a coordinated protein–metabolite regulatory network underlying splenic immune enhancement.

Based on the existing literature, F2R (PAR1) acts as a membrane signaling node linked to G-protein-mediated immune activation and NF-κB signaling [29,30,31]. The observed upregulation of F2R in this study is suggestive of enhanced macrophage activation and lymphocyte proliferative signaling, consistent with elevated IL-2 levels; however, direct mechanistic validation was not performed herein. PLA2G6 participates in phospholipid homeostasis, and its dysregulation has been associated with aberrant immune cell apoptosis and immune dysfunction [32,33,34,35,36]. The significant downregulation of PLA2G6 in the FO group may favor reduced immune cell loss and improved splenic cellular stability, although this interpretation remains inferential. PIGN contributes to genomic stability and metabolic homeostasis [37]; its upregulation may support immune cell integrity, yet post-transcriptional regulation warrants further investigation to clarify protein-level function [38,39]. MGAT5 mediates protein glycosylation that modulates T-cell receptor stability and immune synapse formation [40,41]; its elevated expression implies improved T-cell-dependent cellular immunity, although direct functional evidence is lacking.

Among metabolites, adenosine and hypoxanthine are core components of purine metabolism. Reported functions include immunoregulatory and anti-inflammatory roles for adenosine, and nucleotide biosynthetic support for hypoxanthine. Their concurrent elevation in this study suggests a potential role in maintaining immune homeostasis and mitigating metabolic disturbance-driven immune impairment. Nevertheless, causal links between FO intervention and purine metabolite dynamics remain to be directly validated in this experimental system.

By integrating phenotypic, physiological, and multi-omic data, this study establishes a mechanistic framework wherein FO supplementation enhances splenic immune function by modulating metabolic pathways, calcium signaling, and purine metabolism, accompanied by altered expression of key immune-related proteins and metabolites. These findings advance understanding of plant-based immunomodulants in ruminants, identify candidate molecular targets for functional feed development, and provide a methodological reference for multi-omic studies in nutritional immunology.

Notably, mechanistic interpretations in this study are correlative and literature-supported, rather than causally validated. The proposed roles of F2R, PLA2G6, Calcium signaling, and Purine metabolism are grounded in published evidence but lack direct in vitro or in vivo functional confirmation within this study. The constructed protein–metabolite interaction network serves as a hypothesis-generating tool, not a quantitatively validated regulatory cascade. Accordingly, all mechanistic conclusions are regarded as exploratory and predictive, and future studies employing pathway intervention, gene manipulation, or cell-based functional assays will be necessary to establish causality.

## 5. Conclusions

This study provides correlative multi-omic evidence that dietary fermented onion (FO) remodels the spleen proteome and metabolome in Liangshan Black Sheep, with molecular signatures converging on calcium signaling and purine metabolism—pathways closely associated with enhanced splenic immune function. The candidate immunomodulatory molecules identified, including F2R, PLA2G6, PIGN, SCLY, MGAT5 and metabolites adenosine, hypoxanthine, spermidine, spermine, represent promising targets for developing natural immune-enhancing feed additives for ruminants.

Notably, these findings are associative rather than causally validated. Key limitations include the modest sample size for omics analyses, incomplete protein/metabolite annotation due to limited sheep-specific databases, single-timepoint sampling, and lack of functional validation for the identified pathways and hub molecules. The in silico regulatory network is exploratory and does not confirm direct quantitative interactions.

Future investigations should prioritize larger cohorts, longitudinal sampling, targeted quantitative validation, and in vitro/in vivo functional interventions to establish causality. Standardization of the fermentation process, characterization of bioactive components, and integration of gut microbiome and systemic immune analyses will further refine mechanistic understanding.

Collectively, this work establishes a multi-omic foundation for understanding FO-mediated splenic immunomodulation, supports fermented onion as a sustainable immunomodulatory feed ingredient, and provides a methodological framework for nutrition-immune studies in indigenous livestock breeds.

## Figures and Tables

**Table 1:** Serum immune-related factor concentrations in 6-month-old Liangshan Black Sheep.

Items	Groups	
	CON	T
IgA (mg/mL)	7.86 ± 0.66	10.66 ± 1.29 **
IgG (mgm/L)	14.91 ± 0.57	20.57 ± 1.55 **
IgM (mg/mL)	12.30 ± 1.15	16.98 ± 0.64 **
IL-2 (pg/mL)	838.49 ± 15.95	1068.80 ± 5.66 **
C3 (μg/mL)	600.31 ± 4.11	943.23 ± 7.28 **
SOD (U/mL)	132.84 ± 10.47	216.84 ± 3.79 **
MCP-1 (pg/mL)	14.11 ± 1.10	22.37 ± 1.56 **

Note: *N* = 5; CON represents the control group; T represents the experimental group; the difference in the same row is extremely significant (*p* < 0.01) with the superscript “**”.

**Table 2:** Spleen index of Liangshan Black Sheep fed fermented onion.

Items	Groups		*p* Value
	CON	T	
Body Weight/kg	29.80 ^a^	25.00 ^b^	0.025
Spleen Weight (g)	47.00 ^a^	52.00 ^a^	1.93
Spleen Index (g/kg)	1.58 ^a^	2.08 ^b^	0.025

Note: In the same row, values with different letter superscripts indicate a significant difference (*p* < 0.05); however, with the same or no letter superscripts, it means no significant difference (*p* > 0.05). The same applies below.

**Table 3:** Integrated analysis of differentially expressed proteins and metabolites in spleen tissues of Liangshan black sheep fed with fermented onion and conventional feed.

MapID	MapTitle	Differential Expression Protein Analysis			Analysis of Differential Metabolites in Positive Ion Mode			Analysis of Differential Metabolites in Negative Ion Mode		
		Differential Protein Count	Upregulate Protein	Downregulatory Protein	Differential Metabolites Count	Upregulate Metabolites	Downregulate Metabolites	Differential Metabolites Count	Upregulate Metabolites	Downregulate Metabolites
map04020	Calcium signaling pathway	3	F2R; LOC105614340	PHKG2	1	Adenosine 3′5′-cyclic monophosphate		1	Adenosine 3′,5′-cyclic monophosphate	
map00230	Purine metabolism	4	DGUOK; PGM2	POLD4; PDE10A	6	Adenosine 3′5′-cyclic monophosphate; Adenosine; Hypoxanthine; Adenosine diphosphate (ADP); Adenosine 5′-monophosphate; Inosine		3	Adenosine 3′,5′-cyclic monophosphate; Adenylosuccinic acid	Deoxyinosine
map01100	Metabolic pathways	19	HEXA; NMRK1; DGUOK; PIGN; MTR; DSE; PGM2; GUSB; GALNT12; MGAT5; SCLY, EXT2; LOC105614340	AMDHD1; PLA2G6; POLD4; XYLT1; CPS1; GAPDHS	23	Adenosine 6-Phosphogluconic acid; Creatinine; Hypoxanthine; D-Fructose 1,6-bisphosphate; Spermidine; 5,6-Dihydroxyindole-2-Carboxylic Acid; L-Ascorbate; Adenosine diphosphate (ADP); Adenosine 5′-monophosphate Inosine; PI 38:4; Spermine, L-Tyrosine	Argininosuccinic acid; PC 35:2; Cys-Gly; Palmitic Acid; L-Glutathione (reduced); L-Aspartic acid; PC 32:0; 2-Amino-1,3,4-octadecanetriol; PC 36:1	15	Levodopa; Citraconic acid; Porphobilinogen; Adenylosuccinic acid; Cytidine 5′-Monophosphate-N-Acetylneuraminic Acid; UDP-D-glucuronate; Sedoheptulose 1,7-bisphosphate	Citicoline; N-acetyl-D-glucosamine; 7-Methylxanthine; Deoxyinosine;Dulcitol, dCDP; LPA 20:4; N-Acetylneuraminic acid
map04072	Phospholipase D signaling pathway	2	F2R; DNM2		1	Adenosine 3′5′-cyclic monophosphate		2	Adenosine 3′5′-cyclic monophosphate	LPA 20:4

## Data Availability

The original contributions presented in this study are included in the article. Further inquiries can be directed to the corresponding authors.

## Supplementary Materials

The following supporting information can be downloaded at: https://www.mdpi.com/article/10.3390/vetsci13050486/s1, Figure S1: Results of protein sequence identification of spleen from Liangshan black sheep using DIA technology.
