# Supplementary figures and images for: Integrated Proteomics and Metabolomics Analysis Reveals Potential Pathways Underlying Onion-Mediated Regulation of Spleen Immune Function in Liangshan Black Sheep
Source: Vet Sci. 2026 May 17;13(5):486. doi: 10.3390/vetsci13050486 (PMC13211525; doi:10.3390/vetsci13050486)

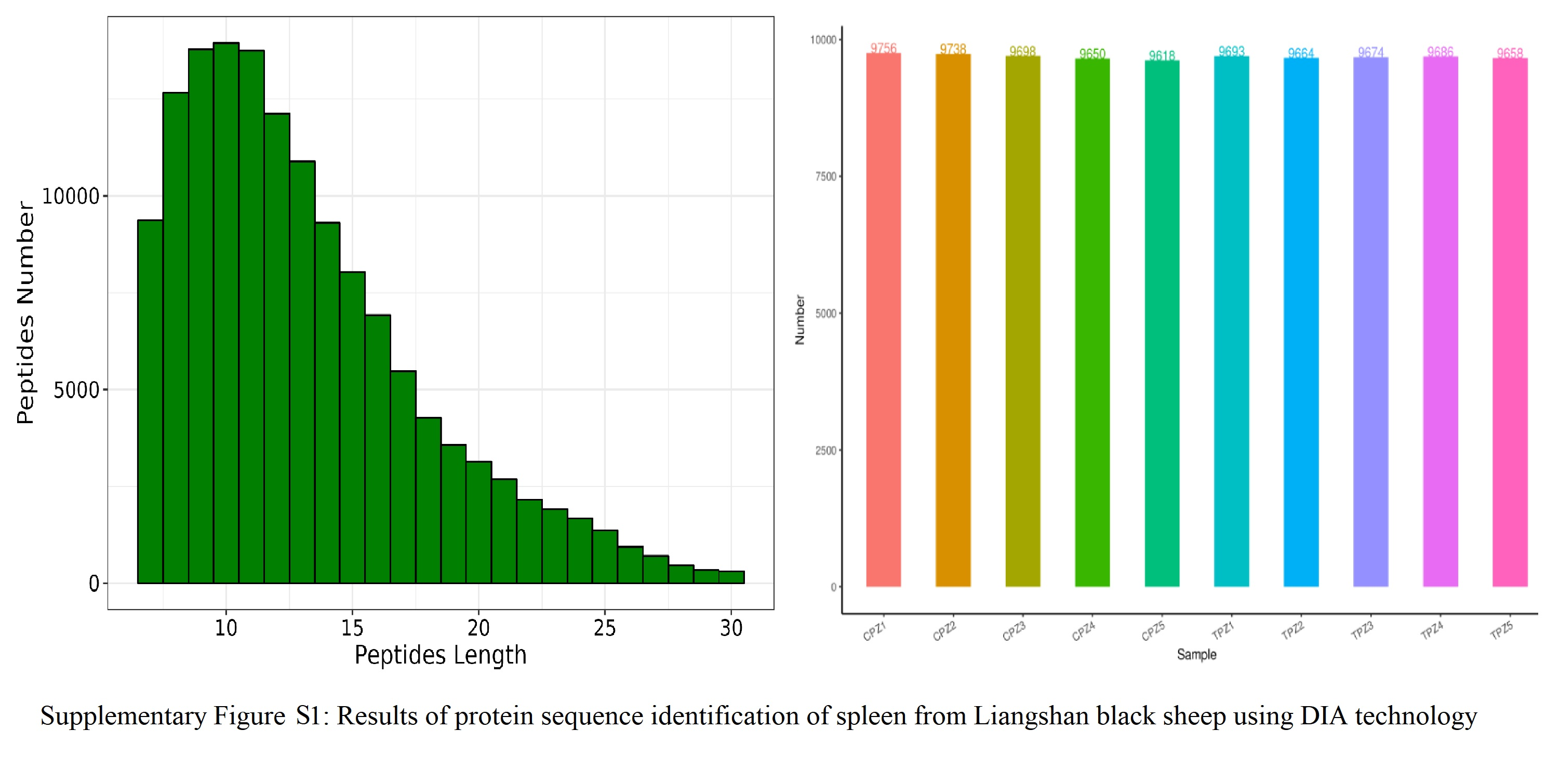

Supplement: Supplementary file 1 [file vetsci-13-00486-s001.zip › vetsci-4238357-supplementary.tif]
